# An Observation of a Very High Swelling of Bromovirus Members at Specific Ionic Strengths and pH

**DOI:** 10.3390/v15102046

**Published:** 2023-10-04

**Authors:** Xochitl Fabiola Segovia-González, Maria Veronica Villagrana-Escareño, Maricarmen Ríos-Ramírez, Vianey Santiago de la Cruz, Jessica Nathaly Mejía-Hernández, Jose Luis Cuellar-Camacho, Araceli Patrón-Soberano, Richard Sportsman, Jaime Ruiz-García

**Affiliations:** 1 Biologycal Physics Laboratory, Physics Institute, Universidad Autónoma de San Luis Potosí, San Luis Potosí 78000, Mexico; xochitl.guao@gmail.com (X.F.S.-G.); veronica.villagrana@gmail.com (M.V.V.-E.); marir87@gmail.com (M.R.-R.); bqvianeysantiago@outlook.com (V.S.d.l.C.); jessymehe@gmail.com (J.N.M.-H.); luiscuecam@gmail.com (J.L.C.-C.); 2 Molecular Biology Division, IPICYT, Instituto Potosino de Investigación Científica y Tecnológica A.C., San Luis Potosí 78216, Mexico; araceli.patron@ipicyt.edu.mx; 3 Department of Chemistry and Biochemistry, University of California Los Angeles, Los Angeles, CA 90095-1569, USA; richsportsman@g.ucla.edu

**Keywords:** virus swelling, CCMV, BMV, dynamic light scattering (DLS), atomic force microscopy (AFM), transmission electron microscopy (TEM)

## Abstract

*Cowpea chlorotic mottle virus* (CCMV) and *brome mosaic virus* (BMV) are naked plant viruses with similar characteristics; both form a T = 3 icosahedral protein capsid and are members of the *bromoviridae* family. It is well known that these viruses completely disassemble and liberate their genome at a pH around 7.2 and 1 M ionic strength. However, the 1 M ionic strength condition is not present inside cells, so an important question is how these viruses deliver their genome inside cells for their viral replication. There are some studies reporting the swelling of the CCMV virus using different techniques. For example, it is reported that at a pH~7.2 and low ionic strength, the swelling observed is about 10% of the initial diameter of the virus. Furthermore, different regions within the cell are known to have different pH levels and ionic strengths. In this work, we performed several experiments at low ionic strengths of 0.1, 0.2, and 0.3 and systematically increased the pH in 0.2 increments from 4.6 to 7.4. To determine the change in virus size at the different pHs and ionic strengths, we first used dynamic light scattering (DLS). Most of the experiments agree with a 10% capsid swelling under the conditions reported in previous works, but surprisingly, we found that at some particular conditions, the virus capsid swelling could be as big as 20 to 35% of the original size. These measurements were corroborated by atomic force microscopy (AFM) and transmission electron microscopy (TEM) around the conditions where the big swelling was determined by DLS. Therefore, this big swelling could be an easier mechanism that viruses use inside the cell to deliver their genome to the cell machinery for viral replication.

## 1. Introduction

*Cowpea chlorotic mottle virus* (CCMV) and *brome mosaic virus* (BMV) are naked plant viruses, and both are members of the *Bromoviridae* family. CCMV is a virus that commonly infects *Vigna unguiculata* [1,2], while BMV commonly infects *Bromus inermis* and other grasses and can be found almost anywhere where wheat is grown. It is also one of the few grass viruses that infects *dicotyledonous* plants, such as soybean [3]; however, it primarily infects *monocotyledonous* plants, such as barley and other plants of the *Gramineae* family. The CCMV and BMV viruses are composed of an icosahedral protein capsid that is approximately 28 nm in diameter [4]. Their capsid is constructed by 180 identical protein subunits, which are arranged into 12 pentamers and 20 hexamers, and each protein has a primary structure of 190 and 189 amino acid residues, respectively. Inside the capsid of CCMV is the (+)ssRNA genome, consisting of around 3000 nucleotides. The genome is divided into three parts, RNA-1 to RNA-3, with an additional subgenomic RNA referred to as RNA-4 [5]. The RNA-1 (3171 nt) and RNA-2 (2774 nt) are encapsidated in a single capsid, while RNA-3 (2173 nt) and RNA-4 (824 nt) are encapsidated together. RNA-1 and RNA-2 are thought to be involved in viral replication throughout the encoding of proteins involved in RNA-dependent RNA replication, while RNA-3 has a role in the spread of infection within the plant through the production of a 32-kDa viral movement protein, and RNA-4 encodes the 20-kDa coat protein [6]. On the other hand, BMV has a genome that is divided into three 5′-capped RNAs and a subgenomic one. RNA-1 (3200 nt) encodes a protein called 1a (109 kDa) [7]. RNA-2 (2900 nt) encodes the 2a protein (94 kDa), and the RNA-dependent RNA polymerase is responsible for replication of the viral genome [8]. The dicistronic RNA-3 (2100 nt) encodes for the 3a protein (involved in cell-to-cell migration during infection), and the coat protein, which is expressed from a subgenomic replication intermediate mRNA, is called RNA-4 (900 nt). The 3a and coat proteins are essential for systemic infection in plants but not for RNA replication [9].

In both cases, the CCMV and BMV protein capsids are arranged in a capsid with a triangulation number T = 3, according to the Caspar–Klug theory [9], where T represents the number of asymmetric positions that the capsid protein adopts in the capsid. The surfaces of the viral nanoparticles present a complex and non-uniform pattern of charged, polar, and hydrophobic residues, which can be adjusted by pH. In addition, the sedimentation coefficient of the *bromoviruses* is lowered below 88 S at low ionic strength and with an increase in pH to 7.5 [9]. Bancroft et al. [10] and Incardona and Kaesberg [11] showed a distinct increase in the hydrodynamic radius of CCMV and BMV particles resulting from a radial extension of the protein and RNA as a function of the pH. It was also corroborated that the radius of the BMV particles increased from 134 Å at pH 5.5 to 155 Å at pH 7.35, as determined by neutron small-angle scattering measurements [12].

The native CCMV virus is stable at a pH close to 5 and has a sedimentation coefficient of 88 S. However, when the pH is near 7 with low ionic strength, the viral particle is in its swollen form [13]. The swelling is due to the expansion of the quasi 3-fold axis. Yet, the RNA does not spontaneously release because of the strong interaction force with the proteins, meaning this process can be reversible [1,4]. This occurs when calcium ions are removed [13,14,15].

For BMV and CCMV, the native state is typically observed at a low pH (˂6.0) in the presence of Mg^2+^, but it experiences a profound, though reversible, structural transition as the pH approaches neutrality [16,17]. At very high-salt conditions, ~1 M, and a neutral or somewhat higher pH, the virion disassembles and loses its RNA as the proteins of the capsid become loose [18,19,20].

The proteins of most viruses assemble in an icosahedral or helical symmetry around their genome. This is a self-assembly process that has also been observed only with the protein subunits without their genome [21], and there are more complex assemblies requiring additional viral (or host) factors [22,23,24]. An icosahedron is defined by 20 triangular faces and 12 vertices related by two-, three-, and five-fold axes of rotational symmetry [25]. As a result of the swelling, it has been proposed that 20 Å pores are formed at each of the quasi-threefold axes of the virion [12,26]. CCMV swells in this way, but magnesium ions, if present on the capsid, prevent both the swelling and the consequent ribonuclease sensitivity [13].

Llauro et al. [27] conducted a study to analyze the stability of TBSV-NPs virus particles under various pH conditions and calcium ion chelation. They found that calcium ions play a significant role in this swelling process, affecting two key aspects: the rigidity of the virus and the elastic limit [28].

Understanding the virus capsid swelling is essential, especially for RNA viruses, because it can help us comprehend how a virus disassembles and/or delivers its genetic material within the cells, as this is a crucial step in the virus replication process. Understanding how this process occurs will give us more information about the function of the capsid within the host cell and how it helps in the release of the genetic material protected by the capsid [1]. In this work, the combined effect of pH and ionic strength on capsid swelling of the CCMV and BMV virions was systematically studied using DLS and complemented with AFM and TEM techniques. As mentioned before, it has been reported that the CCMV virion swells approximately 10% at pH ~ 7. Our results show that, in fact, in most cases of pH and ionic strength, the swelling is about 10%, which confirms these results. Surprisingly, we found that there are specific conditions of pH and ionic strength values where the CCMV and BMV virions swell between 20 and 35%, which could give us a better idea of how the virion delivers its genome to the cell’s replication machinery.

## 2. Materials and Methods

The CCMV was obtained from infected California black eye pea (*Vigna unguiculata*) plants and purified by ultracentrifugation, according to Lavelle et al. [21]. The BMV was obtained from infected barley plants (*Hordeum vulgare*) and was purified by ultracentrifugation following the method of Michel et al. [29]. Both purification methods gave a purity greater than 99.5%, and the viruses were kept in suspension virus buffer (0.1 M sodium acetate, 1 mM ethylenediaminetetraacetic acid (EDTA), and pH 4.8) until they were used.

### 2.1. Characterization Methods

#### 2.1.1. Dynamic Light Scattering (DLS)

This is a technique often referred to as photon correlation spectroscopy (PCS) or quasi elastic light scattering (QELS); it is a common, non-invasive technique for measuring particle size and size distribution, typically in colloidal and nano colloidal suspensions. Virus sizes were measured with a Zetasizer NanoZS (Malvern Instruments Ltd., Worcestershire, UK). Samples were prepared by taking aliquots of the purified virus and then equilibrating them by dialysis against a buffer at a given pH and ionic strength for 24 h, using an 8–12 kDa membrane. Virus samples were prepared in a pH range from 4.8 to 7.6, increasing the pH by 0.2 from sample to sample using the appropriate buffers. For a low pH (in the range from 4.8 to 5.8), we used citrate buffers and phosphate buffers for a higher pH (from 6.0 to 7.6) [30]. The virus concentration used was 0.3 µg/µL. At each pH point, the ionic strength was calculated with the following formula, $I=\frac{1}{2}\sum_{i=n}^{n}C_{i}Z_{i}^{2}$, for each buffer and diluted 1/10 in such a way that the initial ionic strength was 0.01. To reach the desired ionic strength, it was necessary to add NaCl, and the buffers were prepared with autoclaved Milli-Q water. The virus particle size was measured at each pH and ionic strength using the dynamic light-scattering apparatus. At least 7 experiments with 3 measurements each and 100 repetitions were made for each sample.

#### 2.1.2. Atomic Force Microscopy

Atomic force microscopy (AFM) images were acquired with a MultiMode V8 SPM NanoScope microscope (Bruker, Santa Barbara, CA, USA) in a liquid cell at room temperature. The pH and ionic strength conditions for which the experiments were performed were determined from DLS measurements. The images were obtained using the ScanAsyst fluid mode with 512 pixels/line settings and a scanning rate of 0.5 Hz. To ensure the adherence of the virus to the mica surface in solution, it was treated by two methods to change the mica surface charge from a negative to a positive charge, so the negatively charged capsid would adhere to the mica surface. In the case of CCMV, a freshly cleaved mica surface was treated with 10 μL of poly-L-lysine solution (Sigma-Aldrich, St. Louis, MO, USA, MW 70–150 kDa) to alter its surface charge, serving as the substrate [31]. Following this, 20 μL of a diluted CCMV solution (1 mg/mL) was applied to the prepared substrate. This was left to incubate for 15 min at room temperature. Excess solution was then gently blotted off, ensuring the sample did not dry, leaving a thin layer. The sample was then placed on the AFM piezo scanner. Here, the liquid cell was assembled and given around 15 min to achieve thermal equilibrium, as observed using a Thermoacoustic Thermometer (TACT).

In the case of the BMV virus particles, a freshly cleaved mica was first suspended in a magnesium acetate solution (33 mM) overnight to change the surface charge from negative to positive, and after this, it was placed on the AFM piezo scanner, and the liquid cell was mounted afterward. The experiments were carried out in the liquid cell, which was filled with the BMV virus solution in phosphate solution at pH 7 and with an ionic strength of 0.1. Subsequently, it was allowed to stand for two hours to allow the BMV virus particles to adhere to the mica surface through electrostatic interactions. Images were also obtained using the ScanAsyst fluid mode with a setting of 512 pixels/line and a scanning rate of 0.5 Hz. The force applied to both samples, CCMV and BMV, was set to the lowest possible value to avoid surface damage. The cantilever had a nominal tip curvature radius of 2 nm, a spring constant of approximately 0.7 N/m, and a nominal resonance frequency of 150 kHz. The AFM analysis was carried out with the measurement of the height of 100 particles; this study was carried out in the equipment system.

#### 2.1.3. Transmission Electronic Microscopy (TEM)

The size and morphology of the virus were also characterized by TEM using a JEM-200CX (JEOL, Akishima, Japan) transmission electron microscope (TEM) at 100 kV equipped with a digital camera (SIA, Duluth, GA, USA). As mentioned before, the samples were prepared under the conditions where big swelling was detected by the DLS experiments, and then the grid was stored in a desiccator overnight. A 6 µL drop was deposited on a copper grid (300 mesh) that was previously coated with parlodion and carbon. After 1 min, the excess sample was removed with a filter paper, and then the sample was negatively stained with uranyl acetate for samples at pH < 6, and for pH > 6, with a phosphotungstic acid solution. The tungstic acid was prepared at the same pH of the sample; this is important because it can be used in samples with a pH greater than 6 [32]. The images were processed using the ImageJ program to obtain the size distribution histograms, which were constructed from the analysis of at least 100 viral particles; each viral particle was measured in two directions.

## 3. Results and Discussion

As mentioned above, the swelling process of a virus is something that has been proposed as a possible mechanism by which the virus delivers its genetic material to the cell translation machinery. To study this phenomenon, a different series of microdialysis was carried out on two different viruses from the same family under different ionic strength and pH conditions, finding quite interesting results.

To evaluate the size for CCMV and BMV, we first used DLS over a pH range of 4.8 to 7.6 and ionic strengths (I) of 0.1, 0.2, and 0.3, and all the virus samples had a concentration of 0.3 µg/µL. It is important to mention that the DLS shows the hydrodynamic diameter of the capsid, which makes the measurement ~2 nm higher than that observed by TEM. This is due to the fact that water molecules are dipolar, which favors their association with negatively charged surfaces, in this case, the capsid surface. The presence of water molecules increases the size of the capsid measured by DLS, while the measurement in TEM is performed in a vacuum.

**Figure 1:**
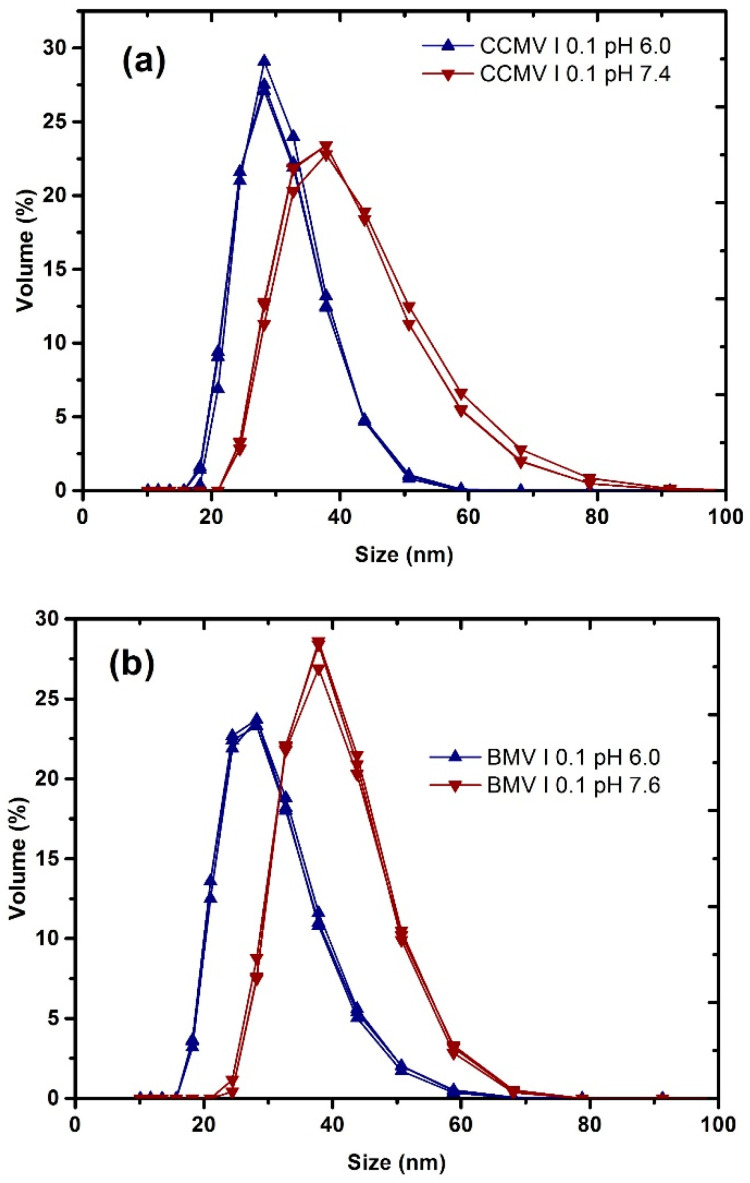
Typical dynamic light-scattering measurements curves of (**a**) CCMV and (**b**) BMB virus particles at I = 0.1 and different pH conditions, where it can be observed that they are different in size due to swelling.

Figure 1a shows three histograms in red for CCMV pH 7.4 and ionic strength of 0.1 and three histograms in blue for pH 6 and the same ionic strength, in which it is clearly seen how the dispersion shifts to the right and the curve broadens at the higher pH, indicating that we have particles of all these sizes and there is a greater polydispersity than when it is found at pH 6. This does not mean that they are individual 65 nm particles as such, as can be seen in Figure S1 of the Supplementary Materials; rather, there are particles joined in the form of doublets in which each particle measures approximately 30 nm. Let us also remember that we are close to the disassembly pH; if the ionic strength is increased in this condition, they could be separated into their components. By maintaining a low ionic strength, it is not possible to break the protein–RNA interactions since, at a pH greater than 7, the predominant or stronger interactions are precisely the protein–RNA interactions. We suggest that, as already mentioned by Speir et al. [4] and Wilts et al. [14], it may be reversible to its native state; however, we do not know if all the RNA remains intact within the capsid. Figure 1b shows a similar trend, but in this case, the dispersion is smaller.

**Figure 2:**
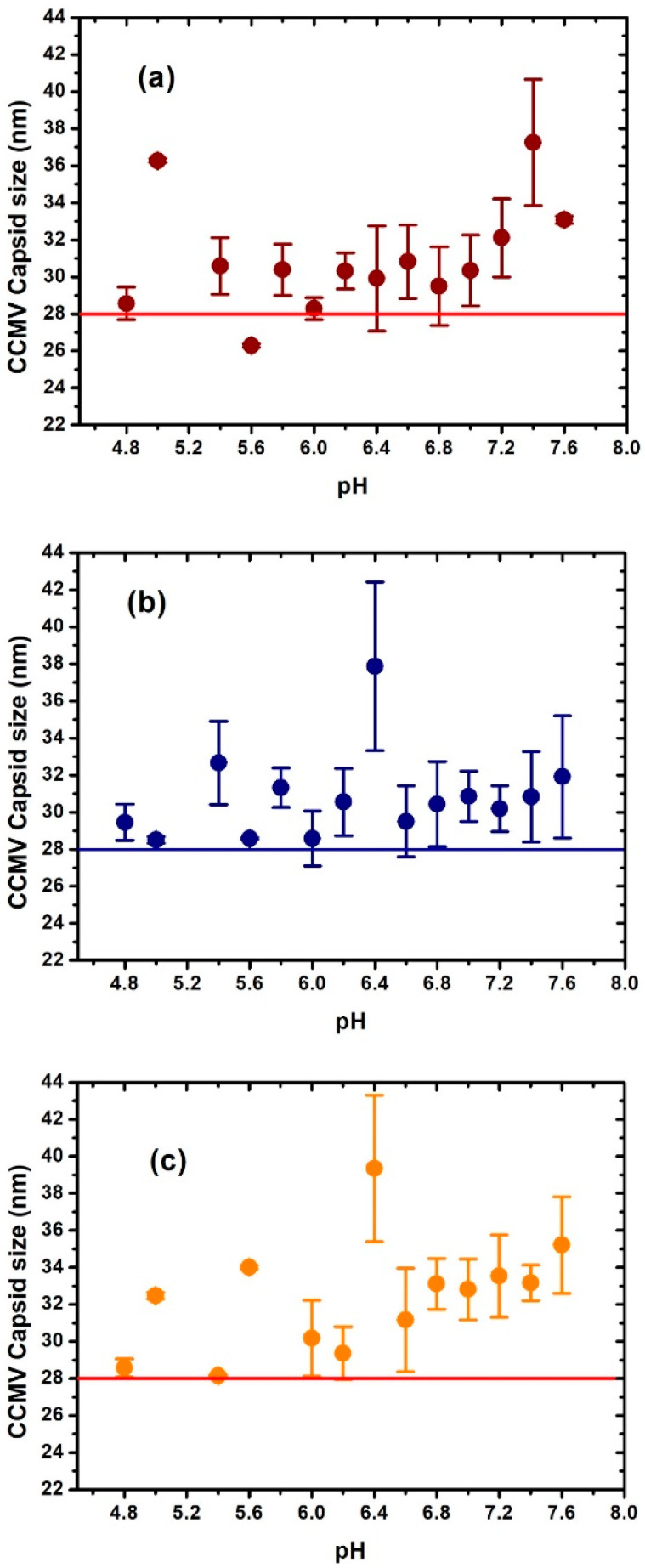
Changes in diameter size of the CCMV virus determined by dynamic light scattering as a function of pH and ionic strength (I). (**a**) I = 0.1; (**b**) I = 0.2; (**c**) I = 0.3.

**Figure 3:**
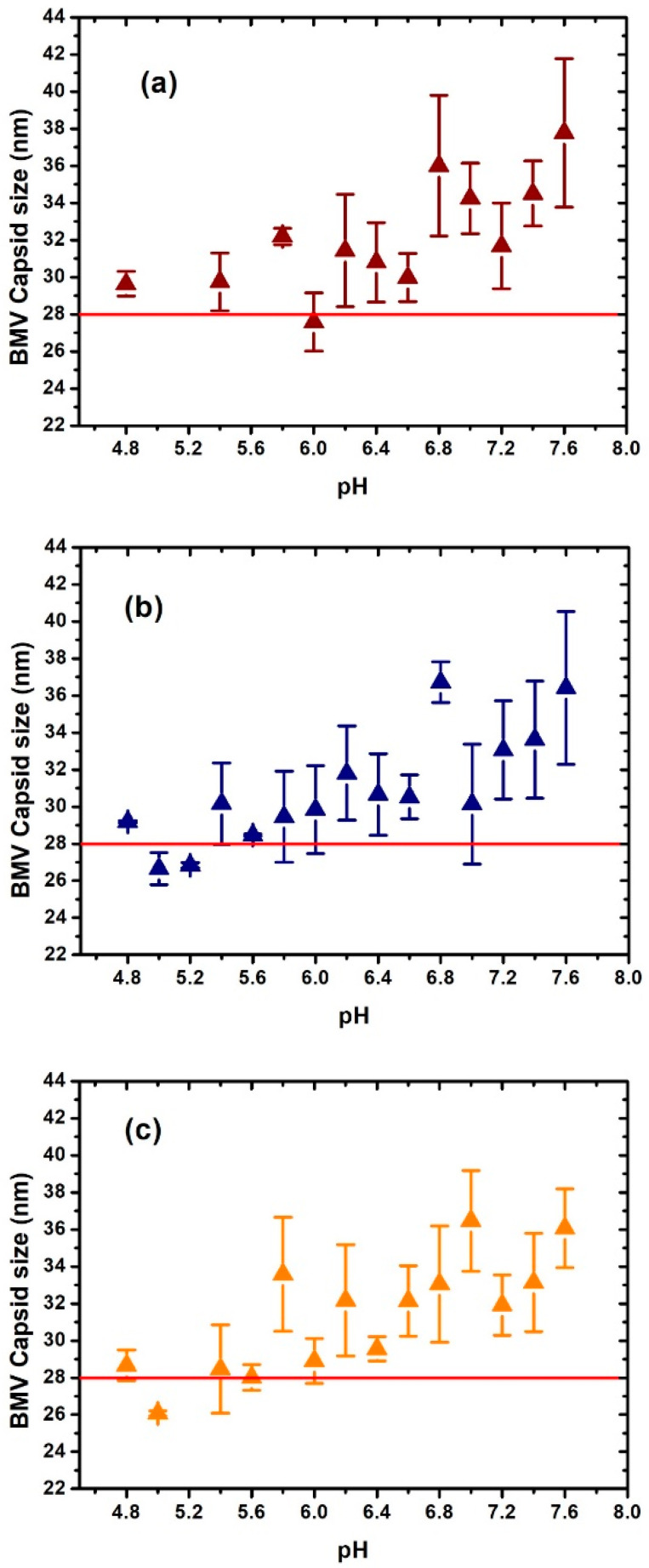
Changes in diameter size of the BMV virus determined by dynamic light scattering as a function of pH and ionic strength I: (**a**) I = 0.1; (**b**) I = 0.2; (**c**) I = 0.3.

Figure 2 and Figure 3 show the difference in size measured by DLS of the CCMV and BMV, respectively, as a function of ionic strength and pH. The horizontal line in each figure is used as a guide to the reported average size for these viruses, considering the virus diameter of around 28 nm reported by TEM. The DLS has standard polydispersity indices (0.05 as a highly monodisperse sample and greater than 0.7 as a sample that is not suitable for the DLS technique). In our case, the polydispersity index of the virus samples is in the appropriate range.

Surprisingly, in Figure 2, we found that in some conditions, very different results were observed for the size of CCMV. For example, at 0.1 ionic strength, a maximum swelling is observed at pH 7.4, where the swelling is ~25% greater than the considered native size of CCMV. At the ionic strength of 0.2, the maximum increase appears at pH 6.4, where the swelling is ~30%. The highest swelling was observed at the ionic strength of 0.3 and at pH 6.4, where the swelling was ~35%. However, note that under other conditions, especially around pH 7, most of the swelling is about 10%, as previously reported.

The evaluation of the swelling of the BMV was carried out in a similar way as the CCMV virion by DLS. The results obtained showed that for an ionic strength of 0.1 and 0.2, the greatest swelling of the virion occurred at a pH of 7.6 and 6.8, respectively, where the swelling was about 25%. For the ionic strength of 0.3, bigger swelling was observed at a pH of 7.0 and 7.6, where the swelling was about 20%. As well as in the case of CCMV, in most cases of pH and ionic strength, swelling was about 10% of the reference value of the virion hydrodynamic diameter.

**Figure 4:**
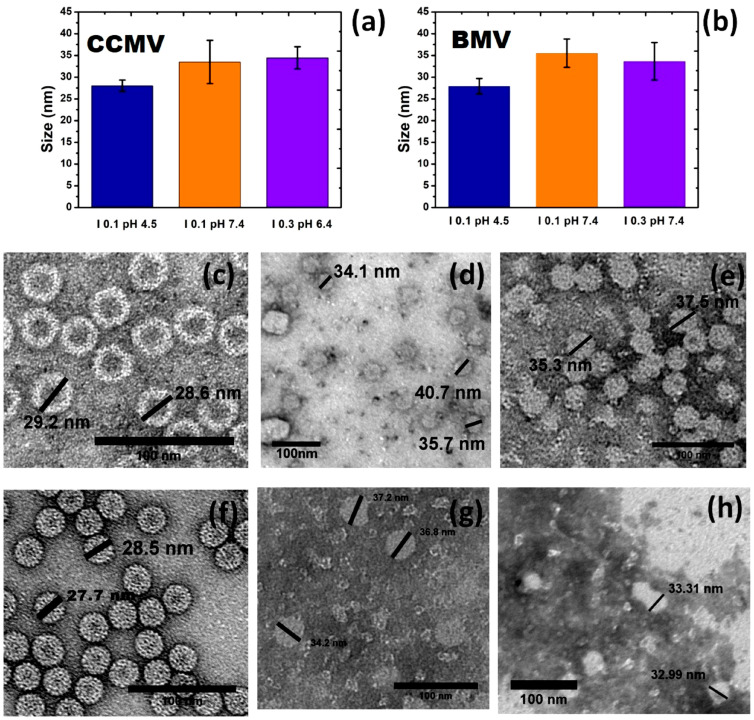
(**a**) CCMV and (**b**) BMV capsid size distribution obtained by TEM at different pH and I conditions; (**c**) CCMV I 0.1 pH 4.5; (**d**) CCMV at I 0.1 and pH 7.4; (**e**) CCMV I 0.3 pH 6.4; (**f**) BMV I 0.1 pH 4.5; (**g**) BMV I 0.1 pH 7.4; (**h**) BMV I 0.3 pH 7.4.

To verify these results, samples were prepared to be observed by TEM under the conditions in which large swelling was detected by DLS and compared to the normal size of the virion, although the TEM results also support that this swelling does not occur in a uniform way. Instead, we see a deformation of the virus capsid, as shown in Figure 4, the images of both the CCMV and BMV virions obtained by TEM. We selected the ionic strength and pH at which the size of the virions is approximately 28 nm and around the conditions where the swelling has its highest value, as determined by DLS. In Figure 4a, the image obtained corresponds to pH 4.5 and I 0.1, which shows a typical image of CCMV and also gives the typical size of the virion of about 28 nm in diameter, while the sizes obtained at I 0.1 and pH 7.4 and I 0.3 and pH 6.4 show a swelling size greater than 20%. In a similar manner, Figure 4b shows the comparison of the normal size, at I 0.1 and pH 4.5, of the BMV virion with the swelling size at I 0.1 and pH 7.4 and I 0.3 and pH 7.4. A swelling greater than 20% for the BMV virion can also be observed. Figure 4c shows images of the normal size of the CCMV virion, and Figure 4d,e show images of the swollen virions at pH 7.4 and I 0.1 and pH 6.4 and I 0.3, respectively. We can observe how the size of the samples under swelling conditions is much larger than the most representative size of the virus samples; furthermore, especially in Figure 4d, some of the capsids are no longer spherical, but they are elongated or deformed. Figure 4f–h correspond to the BMV virus at conditions of pH 4.5 and I 0.1, and pH 7.4 and I 0.1, and I 0.3 pH 7.4, respectively. It can be noted that there is a significant difference in size between the normal accepted size of the BMV virions (Figure 4f) and the BMV swelling virions (Figure 4g,h). In general, the results observed by TEM are consistent with the results obtained by DLS.

**Figure 5:**
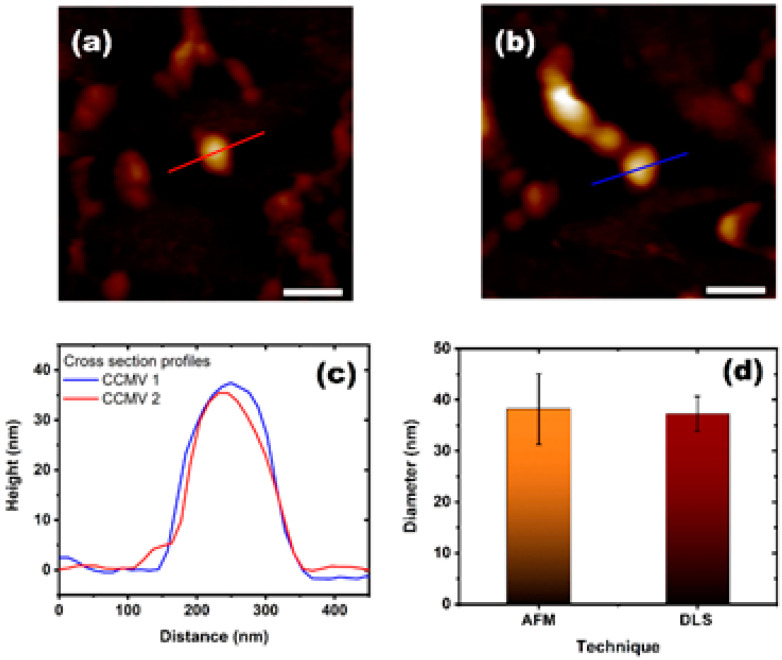
CCMV atomic force microcopy study. (**a**,**b**) AFM images of CCMV viruses in liquid cell at pH 7.4 and I 0.1 in ScanAsyst mode. (**c**) Height profiles of the CCMV virus particles shown in (**a**,**b**) with the lines on them. (**d**). Comparison of the average size of the virus diameter observed by AFM and DLS at the same conditions.

Furthermore, we characterized the swelling of CCMV and BMV virions by AFM in a liquid cell. Figure 5a,b show images of CCMV virions whose height was analyzed at pH 7.4 and I 0.1. In both images, several other particles can be observed, but especially ones that appear extremely high in Figure 5b, which is in line with the measured particle. This particle corresponds to two viral particles, whereby one of them is placed on top of the other, which might be due to the use of the polymer to attach the viral particles to the substrate (see Figure S1 in Supplementary Materials). Figure 5c shows the profile images of the two viruses whose profile lines are indicated in Figure 5a,b. It can be observed that the size is bigger than 35 nm. A comparison between the results of AFM with those obtained with DSL for the same conditions is shown in Figure 5d. We can conclude that the virus has a similar swelling percentage measured by both techniques, with a diameter of 38.2 ± 6.9 nm by AFM and 37.2 ± 3.4 nm by DLS.

**Figure 6:**
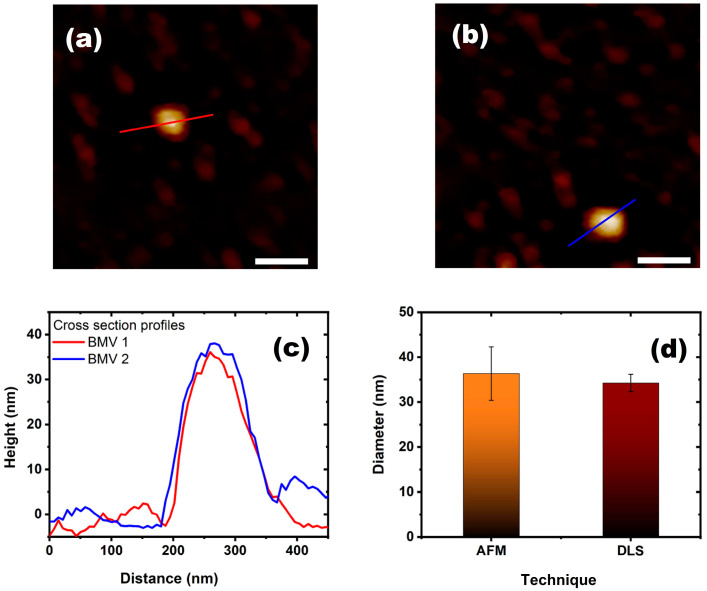
BMV atomic force microcopy study. (**a**,**b**) AFM images of the BMV viruses in a liquid cell at pH 7 and I 0.1 in ScanAsyst mode. (**c**) Height profiles of the BMV virus particles shown in (**a**,**b**) with the lines on them. (**d**). Comparison of the average size of the virus diameter observed by AFM and DLS at the same conditions.

Figure 6a,b show images of two BMV virions at pH 7 and I 0.1. Figure 6c shows the profile images of the two BMV viruses and also shows that the swelling is very similar. In Figure 6d, a comparison between the results of AFM and those obtained with DSL for the same conditions is shown. We can also conclude that the virus has a similar swelling percentage, with a diameter of 36.3 ± 5.9 nm by AFM and 34.2 ± 1.9 nm by DSL.

The experiments in Figure 5 and Figure 6 were carried out for some specific points obtained in AFM in a liquid sample and showed a comparison with very similar values of the data obtained by DLS; therefore, this technique confirms that the data obtained by DLS are reliable.

These results were compared with those of Wilts et al. [14], where they used a phosphate buffer with a molarity of 0.02, but they added PBS, which would increase the ionic strength and impose a difference with respect to our studies. In addition, they also used EDTA, which is a component that is not used in these results. When we repeated these experiments, as performed by Wilts et al. (see Figure S3), we observed that the behavior was very similar and the size difference was approximately 2 nm due to the different measurement techniques. For this reason, we suggest that EDTA plays an important role in the stability of the virus, resulting in less swelling.

It was possible to verify that the CCMV and the BMV present a change in their capsid size when exposed to different conditions of I and pH, resulting in the swelling of the capsid. In addition, we have found that there are specific pH levels and ionic strength conditions under which much greater swelling occurs than reported before. The structural homology of BMV and CCMV capsids is almost identical, but there is a significant difference in the amino acid sequences. Approximately 70% of the sequence is conserved at the protein level. While the beta barrel jelly roll fold is present in many capsids, the bromovirus capsid structure appears to have evolved from other types of capsids based on structural homology. It is possible that BMV and CCMV capsid proteins have a higher structural similarity to kexin-like proteases, unlike other capsid proteins found in nature, and specifically the P domain of these proteases [33]. Therefore, it is possible that the protein structure difference is responsible for the significant swelling change in the BMV virus.

In fact, a key question is whether, given their reversibility, the capsids remain intact with their RNA intact or whether, upon reversion, the virus undergoes changes or loses the RNA. We attempted to address this question using the retardation gel in Figure S2. In this case, a buffer of 0.1 M sodium acetate and 0.001 M EDTA at pH 4.8 was used. The samples loaded onto lanes 2, 3, 5, and 6 were previously dialyzed for 24 h in a phosphate buffer at pH 6.8 and 7.6 for CCMV and BMV, respectively (where the most pronounced swelling was noted based on our data). Lanes 1 and 4 represent the native CCMV and BMV, respectively, placed in the buffer and showing maximum stability at pH 4.5. The reason for the opposite migration of BMV to CCMV is due to its isoelectric point, as reported by Duran-Meza et al. [34], where it was shown that it does not matter if the samples were dialyzed before in certain buffers at different pH levels; the only thing that matters is the pH of the gel buffer where the sample was run. Also, in the work by Miao et al. [35], they mention that the swelling process is reversible, so the gel in Figure S2 was made in order to see if there are RNA losses that would be reflected in a smaller band size (qualitatively), as the gel is stained with GelRed, which is used to visualize nucleic acids. However, no significant changes were observed, indicating that the virus sizes are the same and that it might not be RNA loss.

We assume that in the Wilts et al. [14] experiments, using 1 mM EDTA is not enough to quench all the calcium ions from the capsid protein, and therefore, there is no considerable swelling. On the other hand, in our experiments, we did not use EDTA, as mentioned above. However, we consider that there is a competitive interaction between the sodium salts that we add when calcium and magnesium are present. Remembering that sodium has a larger atomic radius than calcium and magnesium and therefore a higher electron affinity, perhaps the sodium ions are displacing the magnesium ions, and because of that, the level of swelling is much higher. The calcium and magnesium ions give greater stability to protein–protein interactions.

Most vectors transmitting plant viruses are insects, which spread the virus to a new host plant after eating infected leaves and moving to another uninfected plant and eating new leaves [36]. Typically, the solution conditions within the insect gut/mouth are slightly acidic, with a pH from 4 to 6 [37]. The compact (unswollen) conformation of the virion can be visualized by TEM when it contains high concentrations of divalent cations, such as calcium, as the in vitro conditions. After being transported and released into the host cell’s cytoplasm, the capsid experiences a low concentration of free-divalent cations and a pH of 7.4, so capsid swelling is likely to be favored [38]. Different regions within the cell are known to have different pH levels and ionic strengths [39]. It is suggested that under in vitro conditions of pH 7.4 and low divalent cation concentrations, the capsid will swell radially. The diameter may increase by up to 10% with respect to the compact form of the virion, according to previous results [7].

However, we observed a swelling increase much greater than 10 percent for both CCMV and BMV, although BMV appears to swell significantly over a broader pH range than CCMV. It is possible that for BMV, the change is more significant due to the fact that the capsid is less rigid in terms of its structure, more malleable in terms of its form, and more susceptible to changes, in accordance with the experimental studies carried out on BMV [25]. A simple hypothesis, proposed by Wilson et al. [40], is the idea that the viral RNA becomes accessible to the host replication machinery through interactions with the membrane phospholipids, local ionic strength, or pH conditions in the infected plant cell, which might help in removing the first few “unstable” coat protein subunits at the 5′ end, which in this case has been proposed for rod-like viruses, like the tobamo viruses that also infect plants.

From these studies, it has been suggested that ribosomal factors may only require the 5′ end of the RNA, such that the RNA can be extracted from the capsid, which is commonly referred to as co-translational disassembly. The capsid is not required to disassemble immediately upon translation initiation, as has been observed in vitro [41]. Preferential proteolysis of capsid pentamers has been suggested for certain viruses, such as turnip crinkle virus (TCV), to increase viral RNA exposure [42]. Bromoviruses may not require this process, as the capsid shell does not have a strong protein–protein interaction.

## 4. General Conclusions

We show that the CCMV and BMV present high magnitudes of swelling at different ionic strengths and pHs, but more importantly, we show that swelling can be much greater than 10%, as previously reported. It might be that a swelling of around 10% might not be enough for RNA delivery to the cell translational machinery. Whereas a swelling of 20 to 35%, as reported here, could make the viral RNA more accessible for translation. Our work could help better understand how viruses, once inside the host cell, deliver their genetic material to the cellular machinery due to a greater capsid swelling than previously thought, although the detailed mechanisms of this process need more studies.

## Data Availability

Source data are provided with this paper. There is no restriction on data availability.

## Supplementary Materials

The following supporting information can be downloaded at https://www.mdpi.com/article/10.3390/v15102046/s1, Figure S1: Height profile of the particle that looks very high. The profile is about 65 nm, and these high values would correspond to two particles, one on top of the other. Figure S2: Retardation gel run with a buffer of 0.1 M sodium acetate and 0.001 M EDTA at pH 4.8. Samples loaded in lanes 2, 3, 5, and 6 are samples that were previously dialyzed for 24 h in phosphate buffer at pH 6.8 and 7.6 (points at which most swelling was observed) for CCMV and BMV, respectively. Lane 1 and lane 4 correspond to native CCMV and BMV in the highest stability buffer at pH 4.5. The running buffer of the retardation gel has a pH of 4.8. The reason for the migration of BMV to the opposite side of CCMV is due to its isoelectric point, as can be observed in Ref. [34], which would correspond to two particles, one on top of the other. Figure S3: Comparative image between Wilts et al. results performed by AFM and the results obtained with the same conditions in DLS for CCMV and BMV. References [14,34] were cited in the supplementary materials.
