# Supplementary material for: An Observation of a Very High Swelling of Bromovirus Members at Specific Ionic Strengths and pH
Source: Viruses. 2023 Oct 4;15(10):2046. doi: 10.3390/v15102046 (PMC10612077; doi:10.3390/v15102046)
Supplement: Supplementary file 1 [file viruses-15-02046-s001.zip › viruses-2560066-supplementary.pdf]

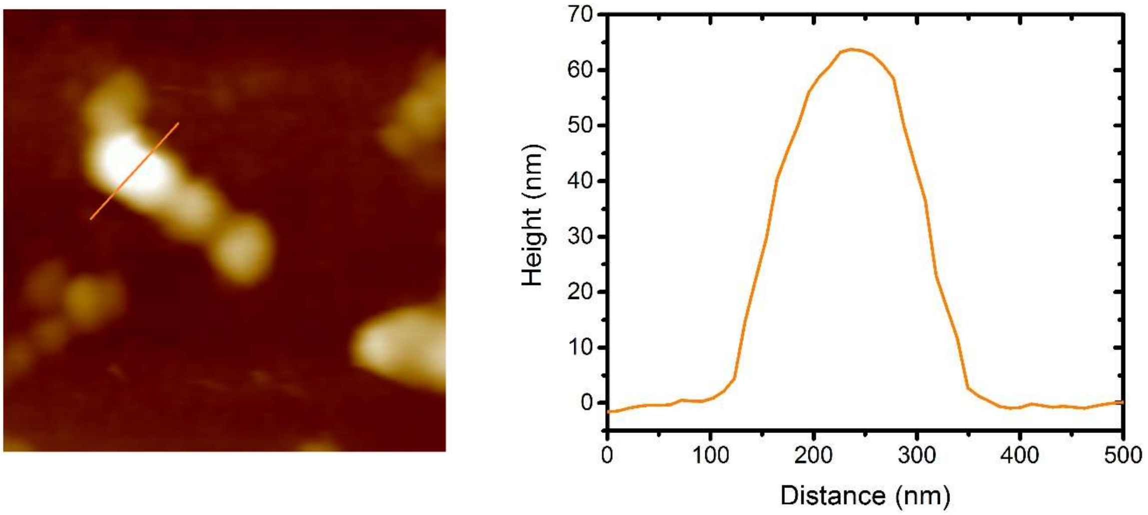

Figure S1. Height profile of the particle that looks very high. The profile is about 65 nm, and these high would correspond to two particles, one on top of the other.

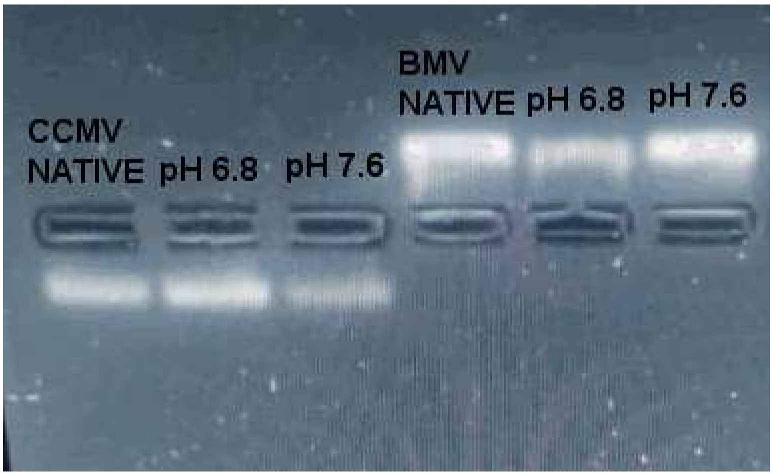

Figure S2. Retardation gel run with a buffer of 0.1 M sodium acetate and 0.001 M EDTA at pH 4.8. Samples loaded in lanes 2, 3, 5 and 6 are samples that were previously dialyzed for 24 h in phosphate buffer at pH 6.8 and 7.6 (points at which most swelling was observed) for CCMV and BMV, respectively. Lane 1 and lane 4 correspond to native CCMV and BMV in the highest stability buffer at pH 4.5. The running buffer of the retardation gel have a pH 4.8. The reason for the migration of BMV being to the opposite side than CCMV is due to its isoelectric point as can be observed with Ref. [34].

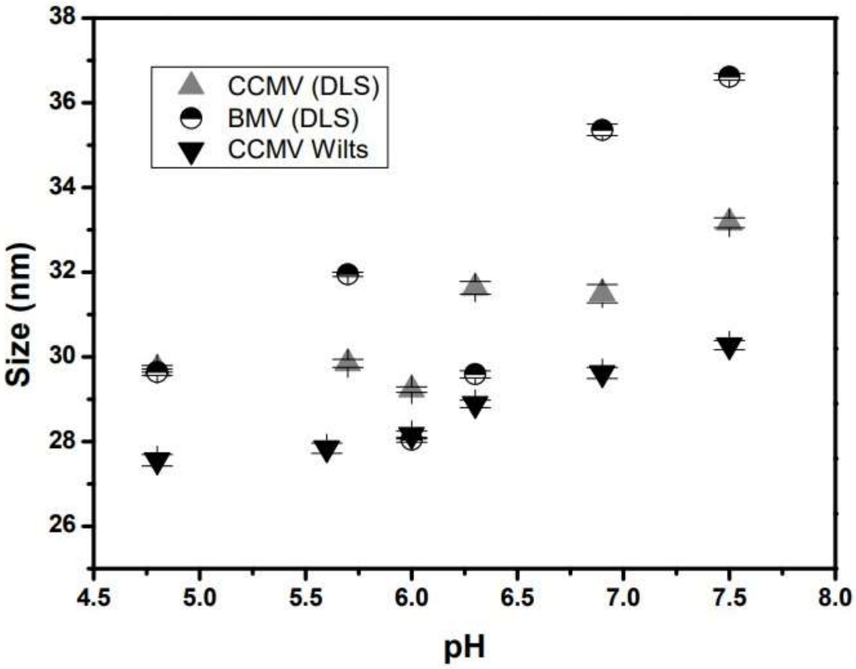

Figure S3. Comparative image between Wilts et al. [14] results performed by AFM and the results obtained with the same conditions in DLS for CCMV and BMV.
